# Ketone Supplements and Alcohol‐Related Responses in Rodents

**DOI:** 10.1111/adb.70079

**Published:** 2025-08-07

**Authors:** Sarah Witley, Sebastian Blid Sköldheden, Christian E. Edvardsson, Elisabet Jerlhag

**Affiliations:** ^1^ Department of Pharmacology, Institute of Neuroscience and Physiology, the Sahlgrenska Academy University of Gothenburg Gothenburg Sweden

**Keywords:** addiction, alcohol use disorder, ethanol, β‐hydroxybutyrate

## Abstract

While alcohol use disorder can be treated with pharmacological interventions, ketosis is a recently proposed treatment option. Ketosis, defined by elevated concentrations of ketone bodies such as β‐hydroxybutyrate (BHB), can be induced by a ketogenic diet or by supplements. As a supplement, both the salt and ester formulation of BHB rapidly increase blood ketone levels. Although preclinical studies have revealed that a ketogenic diet or a mix of ketone supplements reduces alcohol intake and alleviates withdrawal symptoms, the impact of BHB supplements on alcohol‐related responses remains to be defined. We first assessed the efficacy of BHB in ester versus salt formulation on general locomotor activity, exogenous ketosis and alcohol‐induced locomotor stimulation in male mice. We then investigated the impact of the BHB salt on alcohol intake in male and female rats. In attempts to define mechanisms influenced by the BHB salt, monoamines and their metabolites were measured in the nucleus accumbens (NAc), a brain region associated with alcohol reward. Initial results indicate that the BHB salt had a greater impact on ketone levels, glucose‐ketone index and inhibition of alcohol‐induced locomotor stimulation compared to the BHB ester, without altering the general locomotor activity. We further found that BHB salt dose‐dependently lowered alcohol intake in rats of both sexes and that females responded to lower doses than males. Moreover, BHB salt elevated dopamine and noradrenaline and their metabolites in the NAc of male mice. Overall, this study provides insight into the role of BHB salt in modulating rodent alcohol‐related behaviours.

## Graphical abstract

BHB salt and ester both elevate ketone levels. Both ketone supplement formulations prevent alcohol‐induced locomotor stimulation. BHB salt reduces alcohol intake in male and female rats. The image was created using biorender.com.

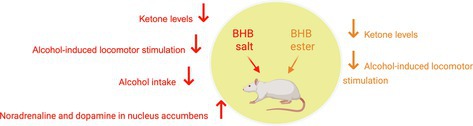

## 1 Introduction

High and prolonged exposure to alcohol contributes to the development of alcohol use disorder (AUD), a complex psychiatric disorder causing both morbidity and mortality [1, 2]. Various pharmacodynamic aspects of alcohol contribute to the risk of developing AUD, with its awarding properties, as measured by the release of dopamine in the nucleus accumbens (NAc), contributing substantially [3, 4]. While pharmacological interventions remain a therapeutic approach for AUD, an alternative proposed treatment is ketosis, defined by elevated blood concentrations of ketone bodies such as acetone, acetoacetate and β‐hydroxybutyrate (BHB). BHB is the most abundant and stable ketone body produced naturally during ketosis [5]. Ketosis can be induced nutritionally through a ketogenic diet, characterized by a high fat and low carbohydrate intake, or through extended fasting [5]. It can also be caused through exogenous ketone supplements, such as BHB salt or BHB ester, which rapidly induce ketosis without a dietary change [5]. Specifically, these exogenous ketone supplements rapidly increase blood BHB levels and maintain them for an extended period, making them highly effective at inducing ketosis [6].

Initial studies revealed that a ketogenic diet decreases alcohol intake in rats [7]; a finding also obtained by intermittent exposure to a high‐fat diet [8, 9]. On a similar note, a ketogenic diet reduces alcohol consumption in male mice without altering the motivation to consume alcohol [10]. In obese AUD patients, a ketogenic diet with low caloric content reduces both alcohol craving and wanting [11]. The impact of a ketogenic diet on alcohol‐related responses is further evident, as it alleviates the withdrawal symptoms in male rats [12], as well as male mice [13]. Similarly, the withdrawal that AUD patients experience during abstinence is improved by a ketogenic diet [7], and the need for benzodiazepines, which are medications used to control alcohol withdrawal symptoms, is reduced [7]. It should, however, be noted that strict adherence to a ketogenic diet is challenging, and exogenous ketone supplements present an alternative by effectively elevating the ketone bodies in the blood independently of carbohydrate restriction [14]. While exogenous ketone supplements have been studied to a lesser extent, a mixture of different ketone esters reduces withdrawal symptoms in abstaining rats [15], and ketone monoester attenuates abstinence symptoms such as convulsions and anxiety‐like behaviours, which are observed during early withdrawal [13]. Moreover, a ketone monoester reduces the number of alcohol‐reinforcing lever presses in male mice [13]. In healthy humans exposed to an alcohol challenge, a mixture of ketone supplements (BHB and R‐1,3‐butanediol) suppresses the liking and wanting for alcohol while enhancing the alcohol dislike [16]. Although these initial studies revealed that a ketogenic diet or exogenous ketone supplements reduce alcohol intake and alleviate withdrawal symptoms, the impact of BHB salt or ester on alcohol‐related responses remains to be defined.

We first assessed the efficacy of ester versus salt formulation of BHB on general locomotor activity and exogenous ketosis in male mice. Given that alcohol causes a robust alcohol‐induced locomotor stimulation in male mice, an effect associated with activation of the mesolimbic dopamine system (for review, see [17]), we evaluated the impact of both formulations on this parameter. We further investigated the effects of different doses of BHB salt on alcohol intake in male and female rats. In attempts to define mechanisms influenced by the BHB salt, monoamines and their metabolites were measured in the NAc, a brain region associated with alcohol reward. Overall, this study provides deeper insight into the role of different formulations of BHB in modulating rodent alcohol‐related behaviours.

## 2 Material and Methods

### 2.1 Animals

In the present study, both adult male mice (NMRI, 25–30 g at arrival; Charles River, Sulzfeld, Germany) and adult male and female rats (Rcc/Han Wistar, 180–250 g at arrival; Envigo, Horst, Netherlands) were used. At arrival, the animals were group‐housed during a week of acclimatization to standardized room conditions (12/12‐h light/dark cycle, 20°C and 50% humidity). The mice were kept group‐housed, and the rats were housed individually, allowing individual intake measurements throughout the experiments. All animals had *ad libitum* access to regular chow (Teklad Rodent Diet, Envigo, Madison, WI, USA) and water. The Ethics Committee for Animal Research in Gothenburg, Sweden (3276/20, 3348/20, 4685/23) approved all experiments, which also followed the ARRIVE guidelines and 3Rs principle.

### 2.2 Drugs

For intraperitoneal (IP) injections, alcohol (95% ethanol, Solveco; Stockholm, Sweden) was diluted in vehicle (0.9% NaCl, 15% [w/v] solution). A dose of 1.75 g/kg, 5 min before the experiments, was used as this dose is known to induce a locomotor stimulation in male mice [18, 19, 20]. For the alcohol intake studies, alcohol was diluted in tap water (20% v/v), as rats prefer this concentration and drink high/stable levels that are intoxicating [21]. Both the salt (D‐(R)‐β‐Hydroxybutyric acid sodium salt (Na‐R‐BHB; Sigma‐Aldrich, Darmstadt, Germany) and ester ((R)‐3‐hydroxybutyl (R)‐3‐hydroxybutyrate; Sigma‐Aldrich) formulations of BHB were dissolved in saline and pH‐adjusted to match vehicle (pH 7.5). Both BHB salt and ester were injected subcutaneously (SC) in a dose range, with the administration route as previously used [22, 23, 24, 25]. To avoid the impact of variation in absorption and metabolism, the peroral administration route was not selected, although this has also been used before [26].

### 2.3 Effects of BHB Ester and BHB Salt on General Locomotor Activity and Exogenous Ketosis in Male Mice

Initial experiments were conducted to evaluate the impact of the ester and salt formulation of BHB on general locomotor activity and exogenous ketosis.

The locomotor activity experiments were conducted in open‐field boxes (42 × 42 × 20 cm; Open Field Activity System, Med Associates Inc., Georgia, VT, USA) that are sound‐attenuated, ventilated and dimly lit (3 lx) as described before [18, 19, 20]. In all experiments, the mice habituated to the test arena for 60 min before drug administration.

In Experiment 1, the male mice were injected with either BHB ester (0.45, 1.5 or 2 g/kg, SC) or vehicle. In Experiment 2, the effects of BHB salt were evaluated; therefore, the male mice were injected with vehicle or a BHB salt dose of 1.5, 2 or 3 g/kg (SC). In both experiments, measurements of the ambulatory distance (cm) started 30 min after injection and were detected for an additional 60 min using a two‐layered grid of infrared photo beams. We also visually inspected the distance data during the first 30 min after injection for both the salt and ester formulations of BHB and observed that higher doses elevated the activity slightly, but to the same extent during this time, and that the activity returned to baseline during this timeframe. These data indicate similarities between the two formulations, and that 30 min between BHB and alcohol injections is suitable. After completing these two locomotor activity experiments, the mice were euthanized (Isoflurane and decapitation), and trunk blood was collected into Eppendorf tubes, and the blood levels of glucose (CONTOUR XT; Ascensia Diabetes Care Sweden AB, Solna, Sweden) were measured when the animals were euthanized. Blood samples were also allowed to clot for 30 min and then centrifuged (10 000 ×*g*) for 10 min to separate the serum. The serum was subsequently collected, and ketone levels in serum (Keto‐Mojo GKI device; Amsterdam‐Duivendrecht, Netherlands) were measured. The glucose‐ketogenic index (GKI), defined as the ratio of glucose to ketone levels, was calculated for each serum sample. To compare the effects of ester and salt, the delta value (% of vehicle–% of treatment) was compared for each of the above parameters.

### 2.4 Effects of BHB Ester on General Metabolism of Alcohol in Male Mice

As BHB ester has been found to alter the metabolism of alcohol after its peroral administration [16], we measured the blood alcohol levels at Sahlgrenska University Hospital as described before [27]. The male mice were injected with BHB ester (0.45, 1.5 or 2 g/kg, SC), and 30 min later, alcohol (1.75 g/kg, IP) was injected. Thirty minutes later, the mice were euthanized, and trunk blood was collected and directly analysed for alcohol levels [27].

### 2.5 Effects of BHB Ester and Salt on Alcohol‐Induced Locomotor Stimulation in Male Mice

Alcohol is well‐known to cause a locomotor stimulation in male mice, and as this effect is associated with activation of the mesolimbic dopamine system, and tentatively to reward, this often serves as an initial test to screen the potential impact of an intervention on alcohol drinking in rodents (for review, see [17]). To investigate the effects of BHB ester and BHB salt on alcohol‐induced locomotor stimulation, three additional experiments (Experiments 3–5) were conducted on male mice. Following a 60‐min habituation, mice received an injection of either BHB ester (2 g/kg, Experiment 3) or salt (2 g/kg, SC, Experiment 4 or 3 g/kg, SC, Experiment 5), or vehicle, followed 30 min later by an injection of alcohol or vehicle. Locomotor activity, measured as ambulatory distance (cm), was recorded starting 5 min after the final injection and monitored for an additional 60 min.

### 2.6 Alcohol Intake Studies in Male and Female Rats

Initial results indicate that the BHB salt had a greater impact on ketone levels, glucose‐ketone index and inhibition of alcohol‐induced locomotor stimulation, compared to the BHB ester, without altering the general locomotor activity. Therefore, the dose‐dependent effect of BHB salt on alcohol intake in male and female rats was investigated in alcohol‐drinking experiments. In these, alcohol intake was measured in the intermittent access two‐bottle choice model, as described in detail before [18, 21, 28]. Briefly, each week, the rats had access to alcohol (20% solution) and water for three 24‐h alcohol‐drinking sessions (Monday, Wednesday and Friday). In the days in between, the rats had access to only water. The bottles were changed at the onset of the dark phase, as rats consume the highest amounts of alcohol during the dark cycle [21, 29]. Male and female rats had access to alcohol for 10 weeks and were thereafter divided into three treatment groups with similar alcohol intake during baseline (data not shown, *n* = 11 per group). Thirty minutes prior to alcohol exposure on the test day, the rats were treated with either vehicle or BHB salt (low doses: 0.5 or 1 g/kg, SC, in Experiment 1; high doses: 2 or 3 g/kg, SC, in Experiment 2). The intake of alcohol, food and water was measured at 4 and 24 h posttreatment, while the rats' body weight was recorded 24 h after treatment. Preset exclusion criteria were leaking bottles, abnormal behaviour or > 15% weight reduction.

### 2.7 Effects of BHB Salt on the Levels of Monoamines and Metabolites in NAc of Male Mice

In attempts to define mechanisms influenced by the BHB salt, monoamines (noradrenaline, dopamine and serotonin) and their metabolites (NM, 3‐MT, DOPAC, HVA and 5HIAA) were measured in NAc using in vivo microdialysis. As described before [18, 19, 20], in vivo microdialysis in combination with HPLC‐EC was used to measure extracellular levels of monoamines and their metabolites in NAc in male mice. After surgical implantation of the probe, the mice recovered for 4 days and were on the test day connected to a microdialysis setup, allowing the collection of 20‐min samples throughout the experiment. Two hours of habituation was followed by a baseline period (−40 to 0 min), and thereafter, the BHB salt (3 g/kg) or vehicle was administered at Minute 0. Eleven additional samples (0–200 min) were collected to determine the treatment impact on NAc neurotransmission. HPLC‐EC was used to detect the levels of monoamines (noradrenaline, dopamine and serotonin) and their metabolites (NM, 3‐MT, DOPAC, HVA and 5HIAA). The changes were calculated as a percentage of the mean of the three baseline values.

### 2.8 Statistical Analysis

GraphPad Prism (version 10.3.0, GraphPad Software Inc., Boston, MA, USA) was used for statistical analyses. A one‐way ANOVA analysed data from the locomotor activity test and intermittent access experiment. An unpaired two‐tailed *t*‐test was used to analyse the delta value comparisons. A repeated two‐way ANOVA was used to analyse data from the in vivo microdialysis experiment. Bonferroni post hoc test was used, and the statistical values were corrected for multiple tests. A *p* < 0.05 was considered statistically significant, and data were presented as mean ± SEM.

## 3 Results

### 3.1 Effects of BHB Ester on General Locomotor Activity, Exogenous Ketosis and Alcohol‐Induced Locomotor Activity in Male Mice

**FIGURE 1:**
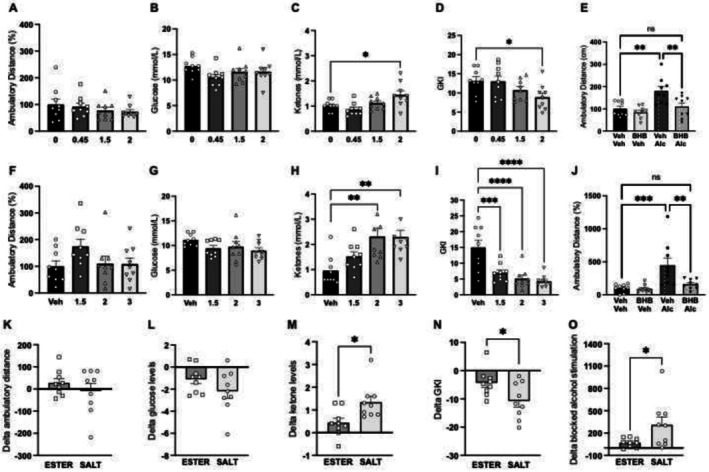
The effects of BHB ester (0.45, 1.5g or 2 g/kg; A–E) and BHB salt (1.5, 2 and 3 g/kg, SC; F–J) on general locomotor activity, exogenous ketosis and alcohol‐induced locomotor stimulation were tested in male mice. (A) Neither of the tested doses of BHB ester altered the distance traveled in male mice. (B) Neither did these doses influence blood glucose levels. (C) BHB ester overall increased the ketone levels in plasma, where the highest dose (2 g/kg, SC) elevated the ketone levels compared to vehicle. (D) BHB ester overall reduced the glucose ketone index (GKI) overall, a reduction observed by the highest dose (2 g/kg, SC). (E) Alcohol caused an alcohol‐induced locomotor stimulation, an effect blocked by BHB ester (2 g/kg, SC). Moreover, mice treated with BHB ester prior to alcohol had a similar activity level as vehicle‐treated mice. Moreover, the selected dose of BHB ester did not alter the distance traveled per se*.* The tested doses of BHB salt did not influence (F) the ambulatory distance or (G) blood glucose levels. (H) In contrast, BHB salt overall elevated the ketone levels. Specifically, the two highest doses (2 and 3 g/kg, SC) markedly elevated the ketone levels. (I) BHB salt caused an overall reduction in GKI, a reduction observed by all three doses. (J) Alcohol caused an alcohol‐induced locomotor stimulation, an effect blocked by BHB salt (3 g/kg, SC). Moreover, mice treated with BHB ester prior to alcohol had a similar activity level as vehicle‐treated mice. Moreover, the selected dose of BHB salt did not alter the distance traveled per se. (K‐O) The delta values (highest dose vs. vehicle) for BHB ester were compared with those for BHB salt to determine which BHB formulation had the greater effect on general activity, exogenous ketosis and blocking alcohol‐induced locomotor activity. (K) The ambulatory distance and (L) blood glucose levels were similar between BHB ester and BHB salt treatments. (M) The BHB salt reduced the ketone levels more than the BHB ester. (N) Similarly, compared to the ester formulation, the BHB salt (N) impacted the GKI more, and (O) blocked the alcohol‐induced locomotor stimulation more profoundly. Data are presented as mean ± SEM. *p < 0.05, **p < 0.01, ***p < 0.001, ****p < 0.0001.

Neither of the tested doses of BHB ester (*n* = 9 per treatment group) altered the distance traveled in male mice (F_3,32_ = 0.79, *p* = 0.5076; Figure 1A) nor did they influence glucose levels (_F3,32_ = 1.93, *p* = 0.1446; Figure 1B). There was an overall effect of BHB ester treatment on ketone levels (F_3,32_ = 5.49, *p* = 0.0037; Figure 1C), where the highest dose (2 g/kg) elevated the ketone levels (*p* = 0.0171), whereas none of the other doses had an effect. Treatment with BHB ester overall reduced the GKI overall (F3,32 = 3.24, *p* = 0.0349; Figure 1D), which is evident as the GKI was lower in male mice treated with the highest dose (2 g/kg) compared to those treated with vehicle (*p* = 0.0315).

In male mice treated with BHB ester (2 g/kg) prior to alcohol (1.75 g/kg), there was an overall treatment effect (F_3,32_ = 8.27, *p* = 0.0003; Figure 1E). Alcohol elevated the distance traveled in vehicle (*p* = 0.0015), but not BHB ester (*p* = 0.9459) pretreated mice. An attenuation in alcohol‐induced locomotor stimulation was further evident, as the alcohol response was lower after BHB ester compared to vehicle (*p* = 0.0060).

### 3.2 Effects of BHB Ester on General Metabolism of Alcohol in Male Mice

Neither of the tested BHB ester doses influenced the blood alcohol levels (F_3,26_ = 0.69, *p* = 0.5656), as the levels of alcohol in blood were similar after vehicle‐alcohol (48.7 ± 0.9, *n* = 7), BHB ester (0.45 g/kg)‐alcohol (48.0 ± 1.5, *n* = 7), BHB ester (1.5 g/kg)‐alcohol (50.1 ± 0.9, *n* = 8) and BHB ester (2 g/kg)‐alcohol (50.3 ± 1.7, *n* = 8).

### 3.3 Effects of BHB Salt on General Locomotor Activity, Exogenous Ketosis and Alcohol‐Induced Locomotor Activity in Male Mice

The tested doses of BHB salt (*n* = 9 per treatment group) did not influence the ambulatory distance of male mice (F_3,32_ = 1.94, *p* = 0.1437; Figure 1F), nor did they affect the glucose levels (F_3,32_ = 1.89, *p* = 0.1520; Figure 1G). Ketone levels significantly increased following BHB salt treatment (F_3,32_ = 6.69, *p* = 0.0012; Figure 1H). Notably, the two highest doses (2 and 3 g/kg) markedly elevated ketone levels (*p* = 0.0019 and *p* = 0.0021, respectively). On a similar note, there was an overall reduction in GKI after BHB salt treatment (F_3,32_ = 12.28, *p* < 0.0001; Figure 1I), which was evident as all three doses of BHB salt reduced the GKI (0.0008, *p* < 0.0001, *p* < 0.0001, respectively).

Two locomotor activity experiments were conducted in which the ability of BHB salt (2 or 3 g/kg) to reduce alcohol‐induced hyperlocomotion was tested. In male mice treated with BHB salt (2 g/kg) prior to alcohol (1.75 g/kg), there was an overall treatment effect (F_3,32_ = 5.32, *p* = 0.0043, *n* = 9 per treatment group). Alcohol elevated the distance traveled in vehicle (*p* = 0.0035, 441 ± 128 cm/60 min for veh‐alc), but not BHB salt (*p* = 0.7005, 193 ± 32 cm/60 min for BGB salt‐alc) pretreated mice. An attenuation in alcohol‐induced locomotor stimulation was further evident as the alcohol response was lower after BHB salt compared to vehicle (*p* = 0.0438, 100 ± 22 cm/60 min for veh‐veh). Compared to vehicle, the selected BHB salt dose did not affect locomotor activity per se (*p* = 0.9987, 123 ± 20 cm/60 min for BHB salt‐veh).

In male mice treated with BHB salt (3 g/kg) prior to alcohol (1.75 g/kg), there was an overall treatment effect (F_3,32_ = 8.04, *p* = 0.0004, *n* = 9 per treatment group; Figure 1J). Alcohol elevated the distance traveled in vehicle (*p* = 0.0007), but not in BHB salt (*p* = 0.8591) pretreated mice. An attenuation in alcohol‐induced locomotor stimulation was further evident, as the alcohol response was lower after BHB salt compared to vehicle (*p* = 0.0052). Compared to vehicle, the BHB salt had no effect on locomotor activity per se (*p* = 0.9999).

The delta values (in % for highest dose vs. vehicle) for BHB ester were compared with those for BHB salt to determine which formulation had the greater effect on general activity, exogenous ketogenesis and blocking alcohol‐induced locomotor activity. The impact of BHB ester compared to salt on ambulatory distance (t_16_ = 0.92, *p* = 0.3750; Figure 1K) and glucose (t_16_ = 0.18, *p* = 0.1782; Figure 1L) was similar between treatments. When comparing the impact of BHB ester and salt on ketone levels (t_16_ = 2.79, *p* = 0.0103; Figure 1M) and GKI (t_16_ = 2.38, *p* = 0.0301; Figure 1N), the salt had a greater effect. Similarly, the ability of BHB to block the alcohol‐induced locomotor stimulation was greater with the salt than with ester (t_16_ = 2.19, *p* = 0.0442; Figure 1O).

### 3.4 Effects of BHB Salt on Alcohol Intake in Male and Female Rats

The effects of different doses of BHB salt were investigated in male and female rats, in which the average 24‐h baseline alcohol intake was 4.4 g/kg for males and 6.9 g/kg for females (both experiments) prior to treatment. While not directly comparable, based on previous literature [21, 30], this may approximately correspond to blood alcohol levels of 10–160 mg%.

**FIGURE 2:**
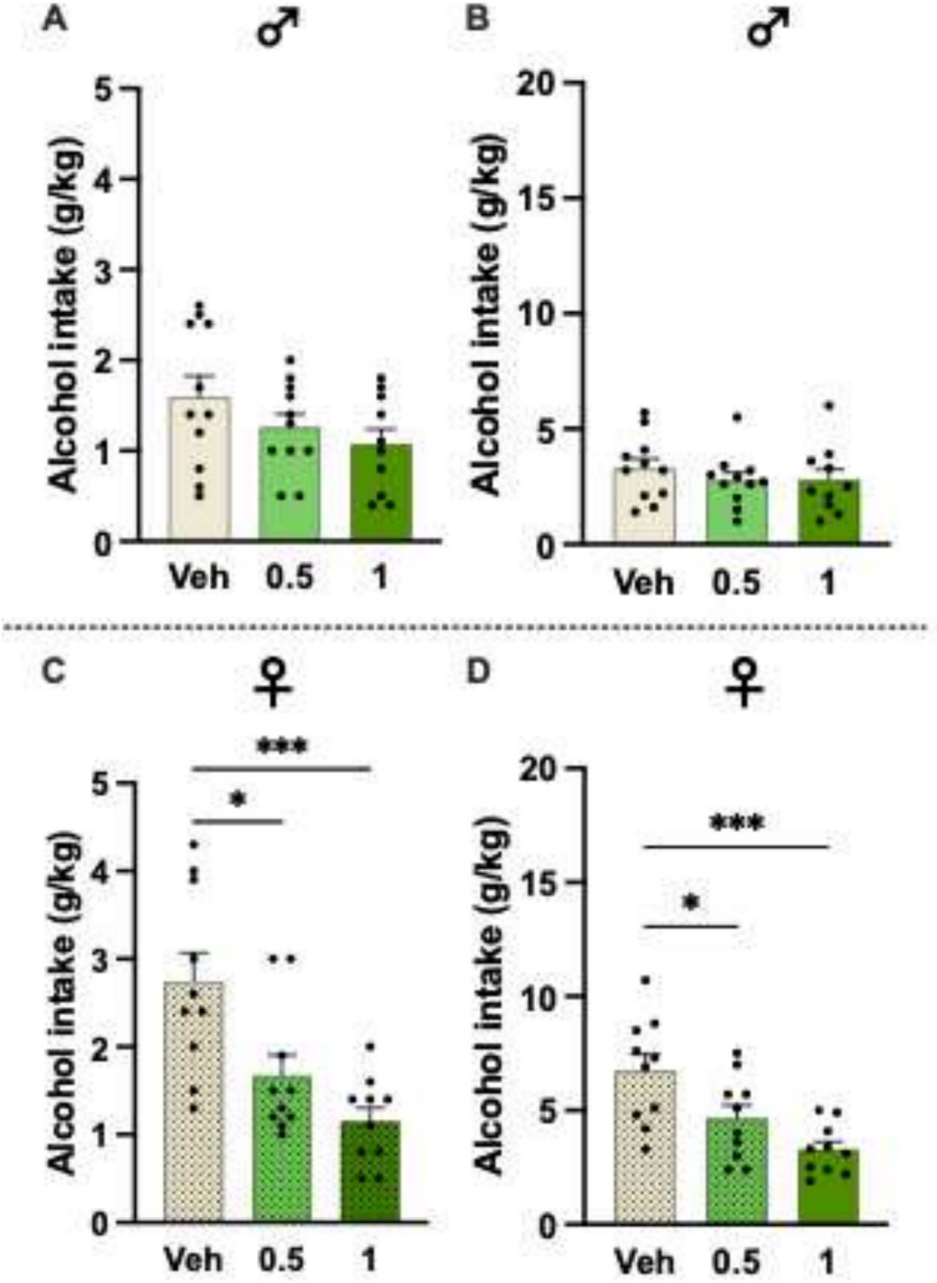
Initial drinking experiments evaluated the effects of low BHB salt doses (0.5 and 1 g/kg, SC) on alcohol intake in male and female rats. In males, BHB salt had no overall effect on alcohol intake at the (A) 4‐ and (B) 24‐h time points. (C) At the 4‐h time point, both BHB salt doses reduced the alcohol intake in females. (D) Similarly, in females, BHB salt overall decreased the 24‐h alcohol intake in female rats, and this reduction was caused by both doses (0.5 and 1 g/kg, SC). Data are presented as mean ± SEM. *p < 0.05, ***p < 0.001.

Initial drinking experiments evaluated the effects of low BHB salt doses (0.5 and 1 mg/kg) on alcohol intake in male and female rats. In males, BHB salt had no overall effect on alcohol intake at the 4‐ and 24‐h time points (Figure 2A,B; F_2,29_ = 1.89, *p* = 0.1692; and F_2,29_ = 0.5220, *p* = 0.5988, respectively, *n* = 11 per group). At the 4‐h time point, BHB salt overall reduced the alcohol intake in females (Figure 2C; F_2,27_ = 10.41, *p* = 0.0004). Compared to vehicle (*n* = 10), both 0.5 mg/kg (*n* = 10, *p* = 0.0106) and 1 mg/kg (*n* = 10, *p* = 0.0002) lowered the alcohol intake at this time point. Similarly, lower BHB salt doses overall decreased the 24‐h alcohol intake in female rats (Figure 2D, F_2,27_ = 9.00, *p* = 0.0010), and this reduction was caused by both 0.5 g/kg (*p* = 0.0313) and 1 g/kg (*p* = 0.0005).

Furthermore, in males, BHB salt treatment overall elevated the water intake at the 4‐h time point (Figure S1A, F_2,29_ = 24.55, *p* < 0.0001). Specifically, the water intake was higher after both 0.5 g/kg (*n* = 11, *p* = 0.0001) and 1 g/kg (*n* = 10, *p* < 0.0001) treatment compared to vehicle (*n* = 11). However, BHB salt did not influence the water intake 24 h after treatment (Figure S1B, F_2,29_ = 1.21, *p* = 0.3128). Neither food intake (Figure S1C,D; 4 h: F_2,29_ = 0.95, *p* = 0.3967 and 24 h: F_2,29_ = 0.47, *p* = 0.6323) nor body weight (Figure S1E, F_2,29_ = 0.78, *p* = 0.4669) was influenced by BHB salt treatment in males.

In female rats, there was an overall increase in water intake by BHB salt treatment at both time points (Figure S1F,G; 4 h: F_2,27_ = 40.22, *p* < 0.0001 and 24 h: F_2,27_ = 8.22, *p* = 0.0016). Specifically, both doses elevated the water intake at the 4‐h (*p* < 0.0001 for both treatments) and 24‐h (0.5 g/kg: *p* = 0.0015 and 1 g/kg: *p* = 0.0015) time points. BHB salt treatment caused an overall reduction in food intake at 4 h (Figure S1H; F_2,27_ = 5.69, *p* = 0.0087) and 24 h (Figure S1I; F_2,27_ = 4.29, *p* = 0.0242) after treatment. At the 4‐h time point, there is a reduction in food intake by both 0.5 g/kg (*p* = 0.0415) and 1 g/kg (*p* = 0.0060) of BHB salt. Twenty‐four hours after treatment, the dose of 1 g/kg (*p* = 0.0132) reduced the food intake. Moreover, BHB salt treatment overall reduced the body weight (Figure S1J; F_2,27_ = 6.166, *p* = 0.0062), where 0.5 g/kg tended to reduce it (*p* = 0.0871), and 1 g/kg significantly reduced the body weight (*p* = 0.0032).

**FIGURE 3:**
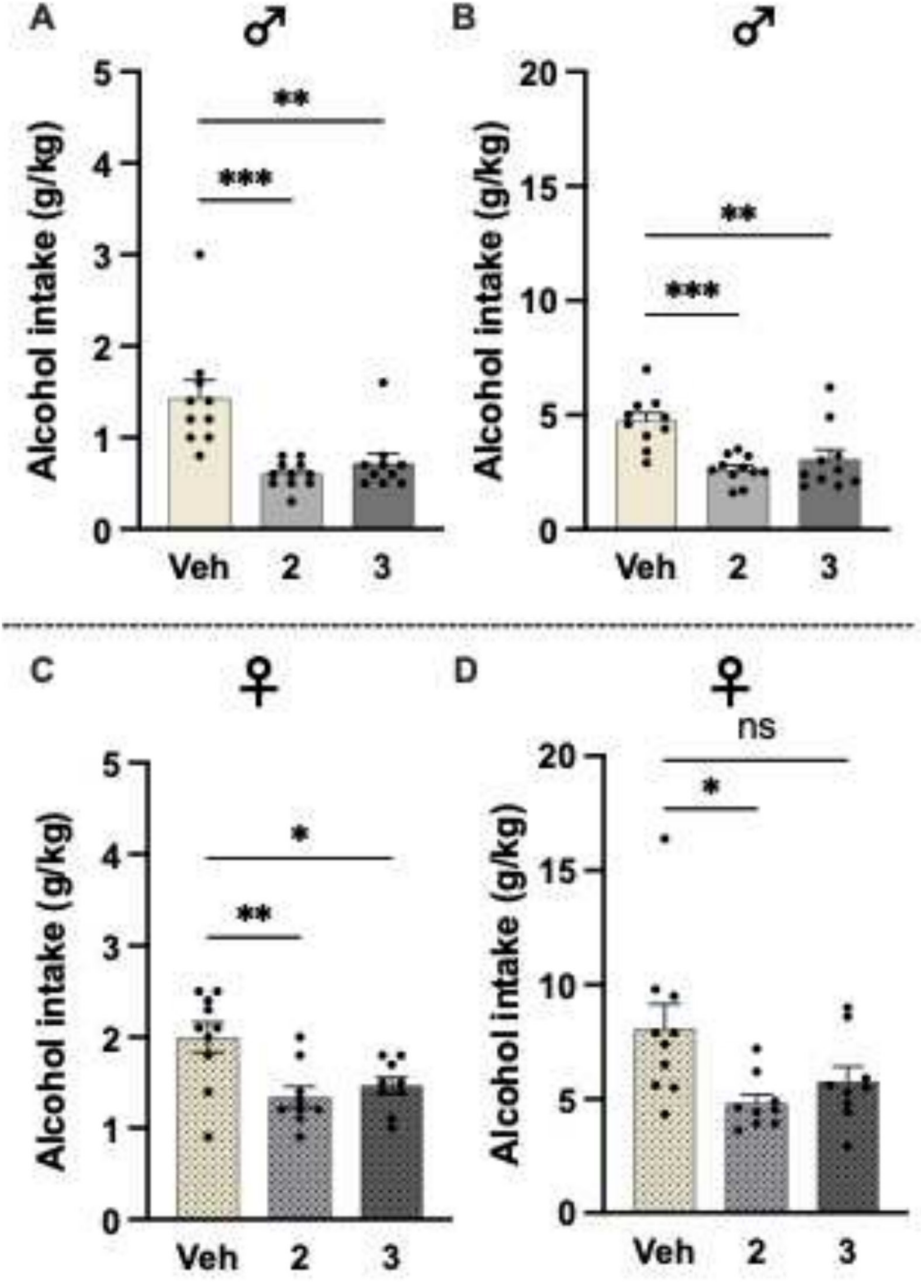
Additional drinking experiments evaluated the effects of higher BHB salt doses (2 and 3 g/kg, SC) on alcohol intake in male and female rats. In male rats, the BHB salt overall reduced the alcohol intake at the 4‐h and (B) 24‐h time points. At both time points, both BHB salt doses lowered the alcohol intake. (C) In female rats, BHB salt overall reduced the alcohol intake at the 4‐h time point, and the alcohol consumption was significantly lowered by both doses. (D) On a similar note, there was an overall decrease in alcohol intake following BHB salt treatment at the 24‐h time point, and this reduction was observed by 2 g/kg (SC). Data are presented as mean ± SEM. *p < 0.05, **p < 0.01, ***p < 0.001.

In male rats, higher doses of BHB salt overall reduced the alcohol intake at the 4‐h time point (Figure 3A; F_2,28_ = 12.50, *p* = 0.0001). Compared to vehicle (*n* = 10), the alcohol consumption was significantly lower following the 2 g/kg (*n* = 11, *p* = 0.0001) and 3 g/kg (*n* = 10, *p* = 0.0010) treatments. On a similar note, there was an overall decrease in alcohol intake following BHB salt treatment at the 24‐h time point (Figure 3B; F_2,28_ = 10.53, *p* = 0.0004), and this reduction was observed by a dose of both 2 g/kg (*p* = 0.0003) and the 3 g/kg (*p* = 0.0037). This overall treatment reduction is further evident in female rats at both 4‐h (Figure 3C; F_2,25_ = 7.34, *p* = 0.0031) and 24‐h (Figure 3D; F_2,25_ = 4.68, *p* = 0.0188) time points. At the 4‐h time point, both 2 g/kg (*n* = 10, *p* = 0.0028) and 3 g/kg (*n* = 10, *p* = 0.0142) lowered the intake in comparison to vehicle (*n* = 11). At the 24‐h time point, there tended to be a reduction of the 3‐g/kg dose (*p* = 0.0869), whereas a dose of 2 g/kg (*p* = 0.0125) significantly lowered the alcohol intake in female rats.

In male rats, there is an overall increase in water intake at both 4 h (Figure S2A; F_2,28_ = 23.12, *p* < 0.0001) and 24‐h (Figure S2B; F_2,28_ = 9.78, *p* = 0.0006) after treatment. This elevation is evident by both 2 g/kg (*p* < 0.0001 at both time points) and 3 g/kg (*p* = 0.00020, *p* = 0.0008, respectively). BHB salt caused an overall reduction in food intake at both time points (Figure S2C,D; F_2,28_ = 13.29, *p* < 0.0001; F_2,28_ = 7.52, *p* = 0.0024, respectively). At both time points, there is a reduction in food intake by the 2‐g/kg dose (*p* = 0.0001, *p* = 0.0005, respectively) and 3‐g/kg dose (*p* = 0.0029, *p* = 0.0064, respectively). On the contrary, there is no effect on the body weight in male rats (Figure S2E; F_2,28_ = 1.30, *p* = 0.2886).

The high doses of BHB salt had a similar effect on water intake, food intake and body weight in female rats as observed in male rats. Specifically, BHB salt overall increased the water intake in female rats at both time points (Figure S2F,G; F_2,25_ = 29.98, *p* < 0.0001 and F_2,25_ = 6.21, *p* = 0.0065 respectively, *n* = 10 per treatment group). Both doses elevated the water intake at the 4 h after treatment (*p* < 0.0001 for both doses). At the 24‐h time point, the water intake was higher in female rats treated with 2 g/kg (*p* = 0.0032), whereas 3 g/kg only tended to alter the water intake (*p* = 0.1532). BHB salt caused an overall decrease in food intake at both time points (Figure S2H,I; F_2,25_ = 7.01, *p* = 0.0038 and F_2,25_ = 4.43, *p* = 0.0226, respectively). Four hours after treatment, both 2 g/kg (*p* = 0.0207) and 3 g/kg (*p* = 0.0031) reduced the food intake. At the 24‐h time point, there was a decline in food intake by 3 g/kg (*p* = 0.0140), but only a trend towards the reduction by the 2‐g/kg dose (*p* = 0.1213). However, there was no effect of the BHB salt on the body weight in females (Figure S2J; F_2,25_ = 0.34, *p* = 0.7116).

### 3.5 Effects of BHB Salt on the Levels of Monoamines and Their Metabolites in Male Mice

**FIGURE 4:**
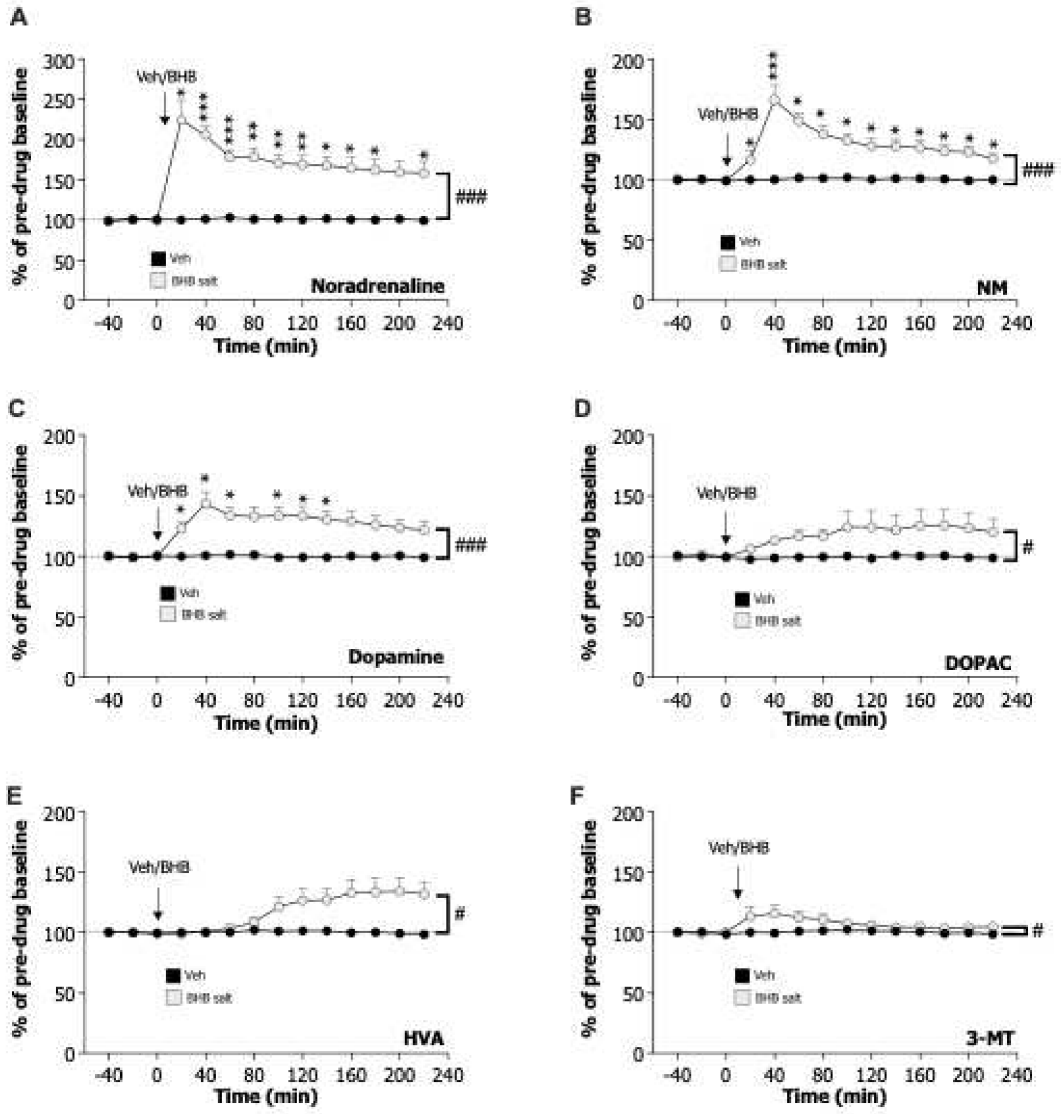
In attempts to define mechanisms influenced by the BHB salt, monoamines (noradrenaline, dopamine and serotonin) and their metabolites (NM, 3‐MT, DOPAC, HVA and 5‐HIAA) were measured in the nucleus accumbens using in vivo microdialysis. Treatment with BHB salt (3 g/kg, SC) increased the levels of (A) noradrenaline and (B) its metabolite, NM. Moreover, the BHB salt elevated (C) dopamine, (D) DOPAC, (E) HVA and (F) 3‐MT. Together, these in vivo microdialysis data demonstrated that BHB salt enhanced the noradrenergic and dopaminergic signaling. Data are presented as mean ± SEM. #*p* < 0.05, ###*p* < 0.001 treatment effect from repeated two‐way ANOVA. *p < 0.05, **p < 0.01, ***p < 0.001 from post hoc test comparing vehicle versus BHB salt at specific time points.

Treatment with BHB salt overall increased the levels of noradrenaline (Figure 4A; treatment F_1,14_ = 40.56, *p* < 0.0001, *n* = 8 per treatment group) as well as its metabolite, NM, in NAc (Figure 4B; treatment F_1,14_ = 39.30, *p* < 0.0001). When it comes to noradrenaline, this increase was evident at time points 20 (*p* < 0.05), 40–60 (*p* < 0.001), 80–140 (*p* < 0.01), 160–180 and 220 min (*p* < 0.05). The levels of NM were higher after BHB salt treatment at time points 20 (*p* < 0.05), 40 (*p* < 0.001) and 80–220 min (*p* < 0.05). Moreover, BHB salt treatment overall elevated dopamine levels in NAc (Figure 4C; treatment F_1,14_ = 23.43, *p* < 0.0001), which was evident at time intervals 20–60 and 100–140 min (*p* < 0.05). In addition, BHB salt caused an overall increase in DOPAC (Figure 4D; treatment F_1,14_ = 5.33, *p* = 0.0367), HVA (Figure 4E; treatment F_1,14_ = 8.51, *p* = 0.0113) and 3‐MT (Figure 4F; treatment F_1,14_ = 6.63, *p* = 0.0220), showing an increased dopaminergic tone by BHB salt treatment. On the contrary, BHB salt did not influence the levels of serotonin (Figure S3A; treatment F_1,14_ = 0.45, *p* = 0.5137) or 5HIAA (Figure S3B; treatment F_1,14_ = 0.02, *p* = 0.8987) in NAc shell.

## 4 Discussion

The present study demonstrated that the BHB salt is the preferred formulation over the ester, as it effectively enhances exogenous ketogenesis without affecting general locomotor activity and inhibits alcohol‐induced locomotor stimulation compared to the ester. Additionally, the BHB salt lowers alcohol intake in rats of both sexes in a dose‐dependent manner, tentatively involving enhanced dopaminergic or noradrenergic neurotransmission in NAc.

Ketone supplements provide a means to achieve ketosis exogenously, defined as elevated ketone levels and lowered GKI [14]. It is widely used as the ketogenic diet requires strict adherence and changes in lifestyle [14]. Here, we demonstrated that BHB salt was advantageous compared to the ester formulation as it significantly enhanced the ketone levels in serum and lowered the GKI. We further revealed that this effect was greater than the one caused by the ester. While neither formulation affects general locomotor activity in male mice, indicating that both forms do not induce behavioural side effects, the salt has a greater ability to block the alcohol‐induced locomotor stimulation in male mice. The findings that BHB salt inhibited the hyperlocomotion caused by alcohol, a marker of stimulation of the mesolimbic dopamine system, indicate that BHB salt suppresses the rewarding properties of alcohol and thereby tentatively reduces alcohol intake, a subject for future studies.

The present study further revealed that the BHB salt dose‐dependently decreased alcohol intake in both male and female rats. While these alcohol‐drinking studies examine the impact of BHB salt on alcohol consumption in both sexes, they are supported by previous research involving ketogenic diets or other ketone supplements in male rodents. Specifically, reduced alcohol consumption has been observed in male rodents following a ketogenic diet [7, 10] or a high‐fat diet [8, 9]. Similarly, the operant self‐administration of alcohol is suppressed by a ketone monoester in male mice [13]. Although not investigated in the current study, ketogenic diets and ketone supplements have been shown to alleviate withdrawal symptoms during alcohol abstinence in male rodents [12, 13, 15]. Future studies should, therefore, investigate the effects of BHB salt on withdrawal symptoms in both male and female rats. Although this study focused exclusively on rodents, the findings may have relevance to humans, as research on ketogenic diets and ketone supplements in rodents has shown translatability to human studies. For example, obese patients with AUD report decreased alcohol cravings and wanting when adhering to a ketogenic diet [11]. Additionally, a ketone supplement has been shown to attenuate the alcohol challenge‐induced enhancement of alcohol liking in healthy volunteers [16]. Similar to findings in rodents, a ketogenic diet reduces withdrawal symptoms in individuals with AUD [7].

The in vivo microdialysis study revealed that BHB salt increased noradrenaline and its major metabolite, NM, in NAc. Moreover, elevated levels of dopamine and the metabolites HVA, DOPAC and 3‐MT were found after BHB salt treatment. Although studies on BHB salt on monoaminergic neurotransmission in NAc are scarce, previous studies have implicated an impact of BHB on dopaminergic signaling. As such, BHB reduces the loss of dopaminergic neurons in an animal model of Parkinson's disease [31, 32]. On a similar note, 3 weeks of ketogenic diet enhance the dopaminergic neurotransmission in the motor and somatosensory cortex of male mice [33]. We further found that the serotonergic pathway was unaffected by the BHB salt treatment, consistent with findings that a ketogenic diet did not alter serotonin or 5HIAA levels in various brain regions [33]. While these data show altered neurotransmission in NAc, the exact underlying mechanisms contributing to the reduced alcohol intake and suppressed activation of the mesolimbic dopamine system remain to be determined. It should be further considered that BHB salt has addictive properties, given its enhanced release of dopamine in NAc, a hallmark of addictive drugs (for review, see [17]). One of these mechanisms could tentatively be gut‐brain peptides as ketogenic diet alters ghrelin, LEAP2 and GLP‐1 [26, 34, 35], which are known to modulate alcohol‐related responses [36].

These alcohol‐drinking experiments reveal sex differences, as higher doses of BHB salt reduced alcohol intake in both sexes, while lower doses decreased alcohol consumption in females without affecting males. One tentative factor that may modulate the sex‐diverging response of BHB is the metabolism of ketones, which is different across sexes [37]. Moreover, the ability of ketone supplements to influence the GKI depends on sex [16]. It should also be noted that sex hormones affect BHB utilization and metabolic pathways, including enzymes central to both pathways [37]. Another tentative factor influenced by sex is the downstream mechanisms of BHB, including the release of GABA or glutamate shown to be altered by BHB [38, 39]. It should be further noted that differences in baseline alcohol intake between the different dose studies might influence the treatment outcome. Since sex‐diverging mechanisms were not examined in this study, future research investigating this topic is warranted.

The present study further showed that low as well as high doses of the BHB salt reduced food intake in females, whereas only high doses lowered feeding in males. These data are in accordance with studies on (R)‐3‐Hydroxybutyrate glycerides and D‐(−)‐3‐hydroxybutyrate, which both have been found to lower food intake in rodents [40, 41, 42]. Supportively, the BHB ester reduces appetite in humans and rodents [26, 43]. In addition, BHB's impact on BDNF and histone methylation is sex‐diverging and has been suggested to influence the lowered food intake observed by BHB [44, 45]. Given that there is no effect on appetite or food intake in human subjects receiving BHB salt perorally [46], the impact on feeding should be investigated in additional animals adapted to food intake. It should be further noted that neither of the tested BHB salt doses acutely influenced the body weight of male and female alcohol‐drinking rats, which is in line with a study on long‐term peroral use of BHB salt, which did not influence body weight and its composition [47]. As previous studies have revealed a body weight reduction and a change in body composition after long‐term BHB salt use [48, 49], future studies with repeated treatments are warranted.

In accordance with the 3R principles, the present study evaluated only the BHB salt—and not the BHB ester—on all alcohol‐related responses, which represents a limitation. A further limitation of the present study is the exclusion of other BHB supplements, such as ketone acids and 1,3‐butanediol, from the experimental design. In the present study, both the ester and salt formulations were given SC, and the effects of oral administrations as tested before [26] g/kg sodium, which in the long run may cause negative health effects including gastrointestinal symptoms, kidney damage and inflammation [50, 51]. It should, however, be noted that long‐term peroral use of BHB salt did not alter the fasting blood safety metrics, bone density, happiness, emotional intelligence or blood pressure in healthy adolescents [47]. Other salt formulations than sodium have been suggested as beneficial [52], and the long‐term effects of these on alcohol intake should be investigated. It should be further noted that pharmacokinetic differences exist between the salt and ester formulations, as the effect of salt may be more long‐lasting but less potent, and this may influence the obtained data. On a similar note, alcohol and BHB ester, but not salt, share the metabolizing pathways, and BHB ester has been found to alter the blood alcohol concentrations [16], another tentative confounding factor. However, we revealed that BHB ester did not influence the blood alcohol levels after their systemic administrations. Peroral administration of BHB ester has been found to lower alcohol absorption [16], a factor that might influence the reduced alcohol intake observed. However, this appears less likely as BHB salt was injected SC, and as it also blocks the stimulatory properties of alcohol when injected IP.

In summary, the present study revealed that BHB salt attenuates the ability of alcohol to activate the mesolimbic dopamine system and reduces alcohol intake in male and female rats. While these novel findings imply a physiological role of BHB, future studies are warranted to define the mechanism of action and the sex‐diverging effects.

## Author Contributions

SW, SBS, and CEE contributed to conceptualization, data curation, formal analysis, methodology, supervision, review and editing, and validation. EJ contributed to conceptualization, data curation, formal analysis, funding acquisition, project administration, resources, supervision, validation, visualization, writing the original draft, and reviewing and editing. All authors met the criteria for authorship and approved the final manuscript, and had access to all data.

## Ethics Statement

The Ethics Committee for Animal Research in Gothenburg, Sweden (3276/20, 3348/20, 4685/23) approved all experiments that also followed the ARRIVE guidelines and 3Rs principle. We are committed to upholding the highest standards of ethics and integrity in all research activities. We strive to conduct research responsibly, transparently, and in compliance with legal, ethical and professional guidelines.

## Conflicts of Interest

The authors declare no conflicts of interest.

## Supporting information


**Figure S1:** In male rats, low doses of the BHB salt (0.5 and 1 g/kg, SC) (A) increased the water intake 4, (B) but not 24 h after treatment. BHB salt treatment did not change the food intake at (C) the 4 or (D) the 24‐h time point. (E) Neither did it influence the body weight of the male rats. In female rats, BHB salt treatment increased the water intake at the (F) 4‐h and (G) 24‐h time points, an increase caused by both doses (0.5 and 1 g/kg, SC). Furthermore, (H) both doses of the BHB salt reduced the food intake 4 h after treatment, (I) and 1 g/kg reduced the food intake 24 h after treatment. (J) Twenty‐four hours after treatment, the dose of 1 g/kg lowered the body weight. Data are presented as mean ± SEM. *p < 0.001.
**Figure S2:** In male rats, higher doses of the BHB salt (2 and 3 g/kg, SC) (A) increased the water intake 4, and (B) 24 h after treatment. BHB salt treatment lowered the food intake at (C) the 4‐ and (D) the 24‐h time point. (E) However, neither does influence the body weight of the male rats. In female rats, BHB salt treatment (2 and 3 g/kg, SC) elevated the water intake at the (F) 4‐h and (G) 24‐h time points. Furthermore, (H) both doses of the BHB salt reduced the food intake 4 h after treatment, (I) and 3 g/kg reduced the food intake 24 h after treatment. (J) However, neither does influence the body weight of the female rats. Data are presented as mean ± SEM. *p < 0.001.
**Figure S3:** In male mice, BHB salt (3 g/kg, SC) did neither influence the extracellular levels of (A) serotonin nor (B) the metabolite, 5HIAA in the nucleus accumbens. Data are presented as mean ± SEM.

## Data Availability

The data will be made available upon request.
