# Supplementary material for: Ketone Supplements and Alcohol‐Related Responses in Rodents
Source: Addict Biol. 2025 Aug 7;30(8):e70079. doi: 10.1111/adb.70079 (PMC12329427; doi:10.1111/adb.70079)
Supplement: Supplementary file 1 — Figure S1: In male rats, low doses of the BHB salt (0.5 and 1 g/kg, SC) (A) increased the water intake 4, (B) but not 24 h after treatment. BHB salt treatment did not change the food intake at (C) the 4 or (D) the 24‐h time point. (E) Neither did it influence the body weight of the male rats. In female rats, BHB salt treatment increased the water intake at the (F) 4‐h and (G) 24‐h time points, an increase caused by both doses (0.5 and 1 g/kg, SC). Furthermore, (H) both doses of the BHB salt reduced the food intake 4 h after treatment, (I) and 1 g/kg reduced the food intake 24 h after treatment. (J) Twenty‐four hours after treatment, the dose of 1 g/kg lowered the body weight. Data are presented as mean ± SEM. *p < 0.001. Figure S2: In male rats, higher doses of the BHB salt (2 and 3 g/kg, SC) (A) increased the water intake 4, and (B) 24 h after treatment. BHB salt treatment lowered the food intake at (C) the 4‐ and (D) the 24‐h time point. (E) However, neither does influence the body weight of the male rats. In female rats, BHB salt treatment (2 and 3 g/kg, SC) elevated the water intake at the (F) 4‐h and (G) 24‐h time points. Furthermore, (H) both doses of the BHB salt reduced the food intake 4 h after treatment, (I) and 3 g/kg reduced the food intake 24 h after treatment. (J) However, neither does influence the body weight of the female rats. Data are presented as mean ± SEM. *p < 0.001. Figure S3: In male mice, BHB salt (3 g/kg, SC) did neither influence the extracellular levels of (A) serotonin nor (B) the metabolite, 5HIAA in the nucleus accumbens. Data are presented as mean ± SEM. [file ADB-30-e70079-s001.pptx]

## Slide 1
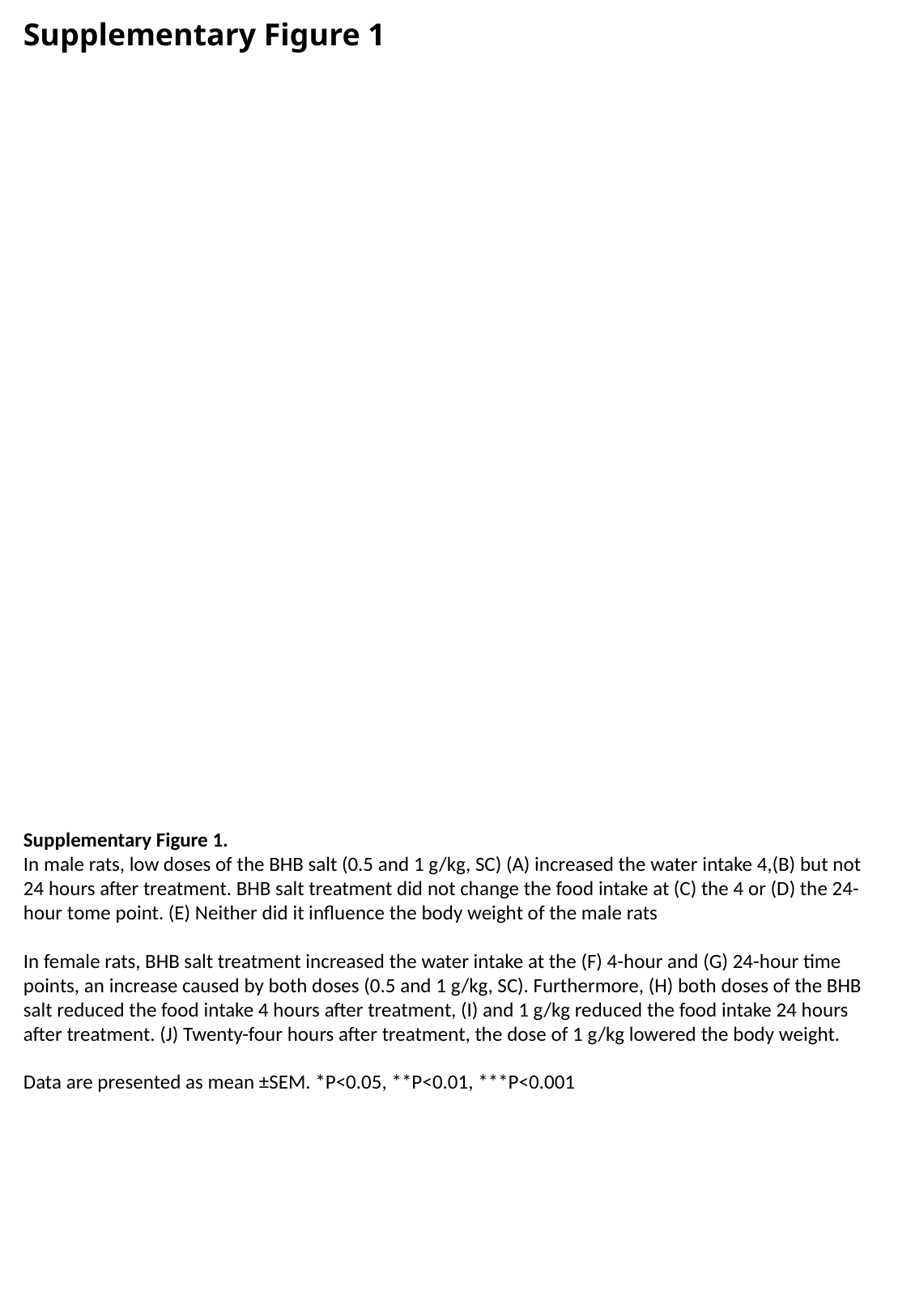

Supplementary Figure 1
Supplementary Figure 1.
In male rats, low doses of the BHB salt (0.5 and 1 g/kg, SC) (A) increased the water intake 4,(B) but not 24 hours after treatment. BHB salt treatment did not change the food intake at (C) the 4 or (D) the 24-hour tome point. (E) Neither did it influence the body weight of the male rats
In female rats, BHB salt treatment increased the water intake at the (F) 4-hour and (G) 24-hour time points, an increase caused by both doses (0.5 and 1 g/kg, SC). Furthermore, (H) both doses of the BHB salt reduced the food intake 4 hours after treatment, (I) and 1 g/kg reduced the food intake 24 hours after treatment. (J) Twenty-four hours after treatment, the dose of 1 g/kg lowered the body weight.
Data are presented as mean ±SEM. *P<0.05, **P<0.01, ***P<0.001

## Slide 2
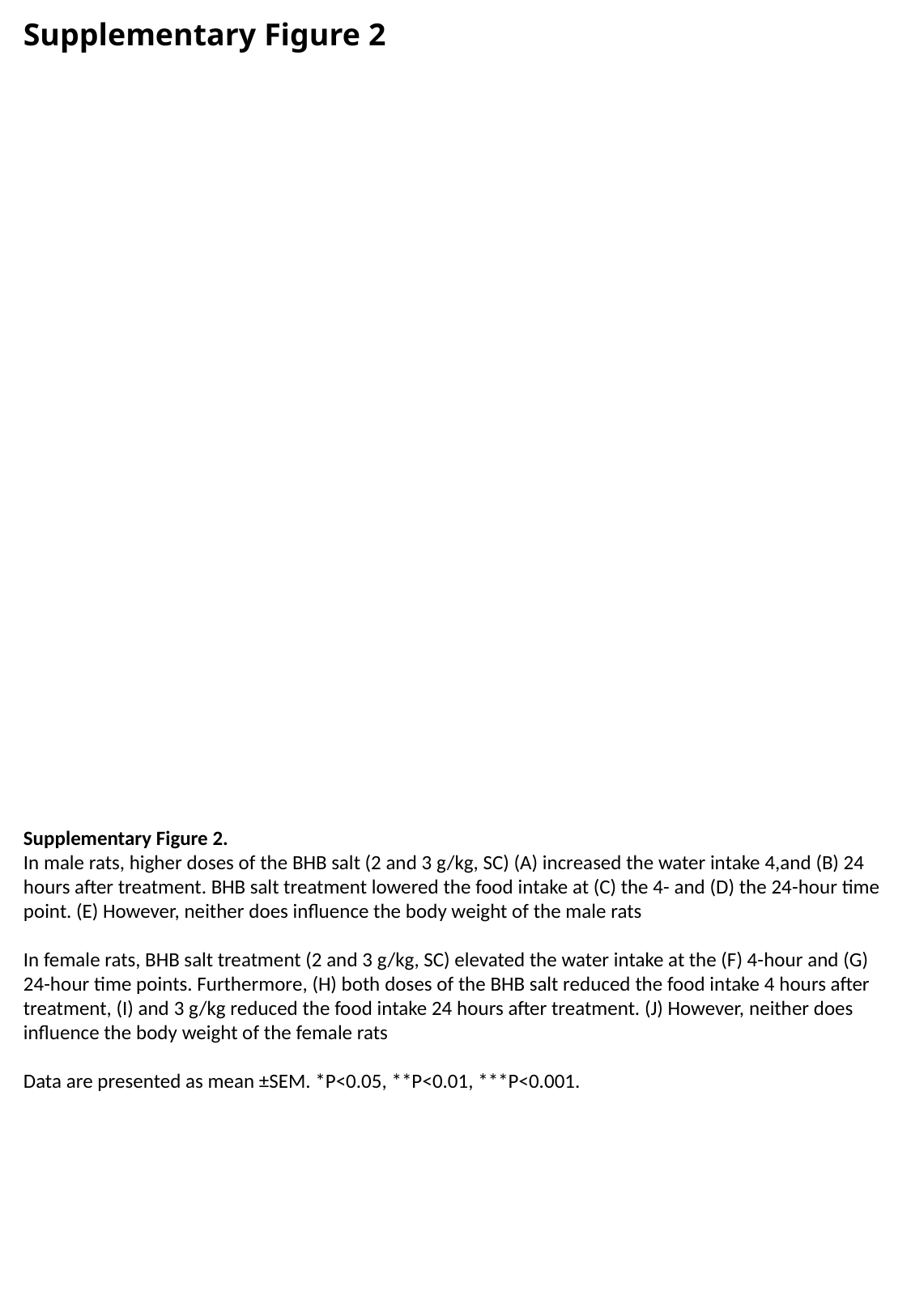

Supplementary Figure 2
Supplementary Figure 2.
In male rats, higher doses of the BHB salt (2 and 3 g/kg, SC) (A) increased the water intake 4,and (B) 24 hours after treatment. BHB salt treatment lowered the food intake at (C) the 4- and (D) the 24-hour time point. (E) However, neither does influence the body weight of the male rats
In female rats, BHB salt treatment (2 and 3 g/kg, SC) elevated the water intake at the (F) 4-hour and (G) 24-hour time points. Furthermore, (H) both doses of the BHB salt reduced the food intake 4 hours after treatment, (I) and 3 g/kg reduced the food intake 24 hours after treatment. (J) However, neither does influence the body weight of the female rats
Data are presented as mean ±SEM. *P<0.05, **P<0.01, ***P<0.001.

## Slide 3
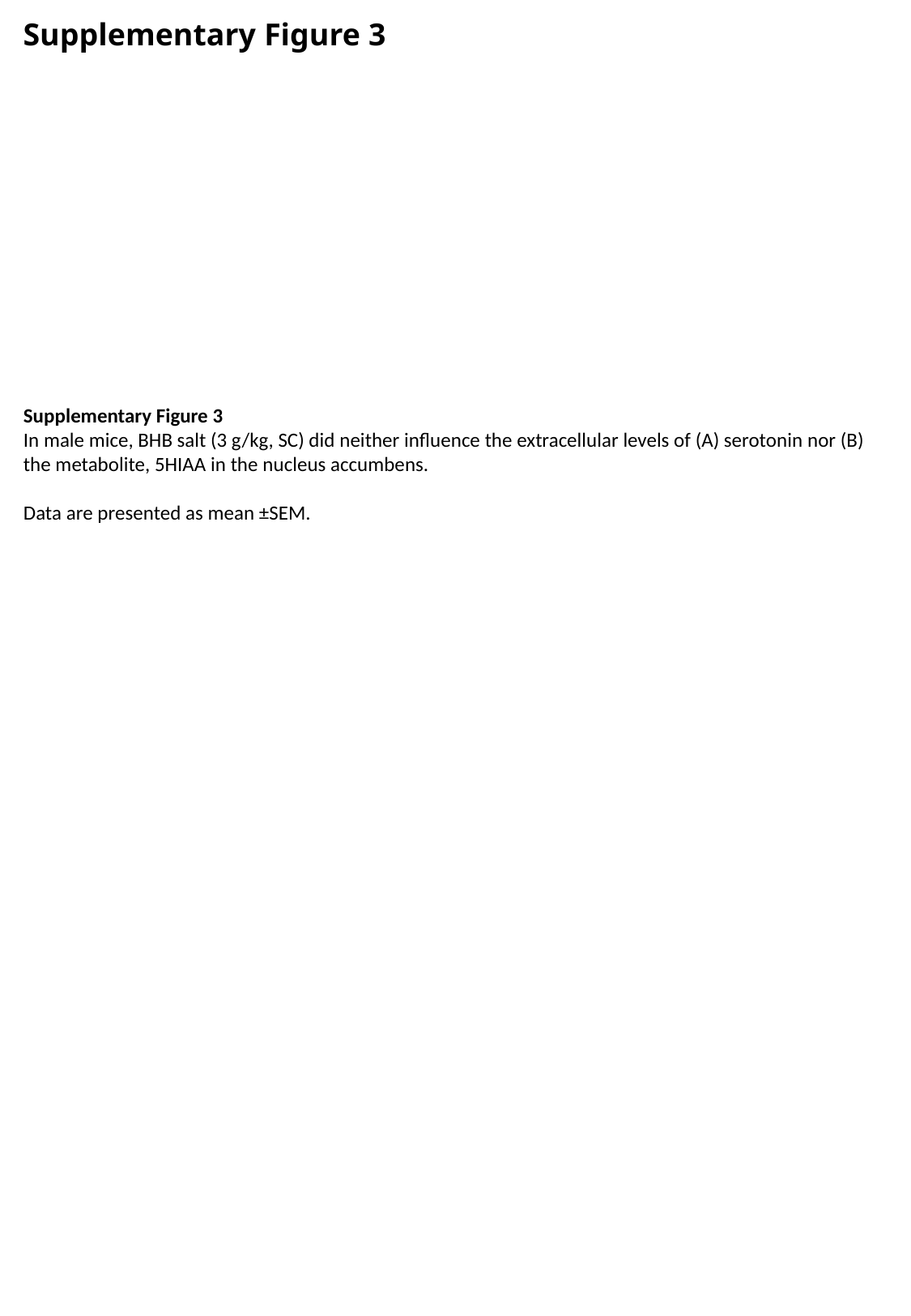

Supplementary Figure 3
Supplementary Figure 3
In male mice, BHB salt (3 g/kg, SC) did neither influence the extracellular levels of (A) serotonin nor (B) the metabolite, 5HIAA in the nucleus accumbens.
Data are presented as mean ±SEM.
